# Three case reports: Temporal association between tyrosine-kinase inhibitor-induced hepatitis and immune checkpoint inhibitors in renal cell carcinoma

**DOI:** 10.1097/MD.0000000000018098

**Published:** 2019-11-22

**Authors:** Alberto Carretero-González, Javier Salamanca Santamaría, Daniel Castellano, Guillermo de Velasco

**Affiliations:** a Department of Medical Oncology; b Department of Pathology, University Hospital 12 de Octubre, Madrid, Spain.

**Keywords:** immune checkpoint inhibitors, hepatic toxicity, renal cell carcinoma, safety profile, tyrosine-kinase inhibitors

## Abstract

**Rationale:**

Hepatotoxicity is a well-known adverse effect of vascular endothelial growth factor receptor (VEGFR) tyrosine-kinase inhibitors (TKIs), usually employed for the treatment of metastatic renal cell carcinoma (mRCC). Immune checkpoint inhibitors (ICIs) have been shown to improve survival in specific patients with mRCC, but concerns have arisen over their safety profile, particularly as regards the risk of liver damage in those patients receiving TKIs sequentially or concurrently with these new drugs. Here, we report three cases of hepatitis presentation in patients receiving TKIs after ICIs that should potentially be considered in current clinical practice, where a combination of these hepatotoxic drugs is becoming increasingly used.

**Patients concerns:**

All three patients were receiving TKIs therapy and presented with nonspecific clinical deterioration and liver enzyme elevation in different time frames according to the start of treatment. All were previously treated with ICIs.

**Diagnoses:**

After performing imaging techniques and complementary laboratory tests for the differential diagnosis of hepatic injury, the diagnosis of potentially TKI-induced hepatitis was assumed in all these cases. Hepatic biopsy was performed only in the first case in order to confirm the diagnosis.

**Interventions:**

Potential toxic drugs were interrupted and steroids course with slow reduction regimen was administered in all these cases because of the previous use of ICIs.

**Outcomes:**

The patients described improved with this conservative treatment without complications during the following weeks. Only one case presented a new episode of mild hepatic alteration while on treatment with following treatment.

**Lessons:**

Taking into account this new therapeutic context, stricter monitoring for potentially increased/altered adverse events should be indicated. Adequate patient selection and consideration of the safety profile of the different drugs used could help to optimize treatment in the near future.

## 1 Introduction

Hepatotoxicity was described as one of the class-related safety issues during the development of vascular endothelial growth factor receptor (VEGFR) tyrosine-kinase inhibitors (TKIs) for the treatment of metastatic renal cell carcinoma (mRCC). Biochemical markers of liver injury include elevated alanine aminotransferase (ALT), aspartate aminotransferase (AST), alkaline phosphatase (ALP) and bilirubin levels.^[1]^

Hepatotoxicity can take several weeks to develop, and often simply manifests as elevated liver enzymes; the latency period after starting therapy to increased ALT levels is 2 to 8 weeks in most cases.^[2,3]^ In clinical trials, hepatitis was commonly observed during TKI treatment in up to 50% of patients, generally transient, and mostly resolved without treatment discontinuation. Approximately 2% to 5% of patients develop high-grade ALT elevation, with or without jaundice.^[3]^. Severe hepatotoxicity varies according to the type of VEFGR-TKI (sorafenib, sunitinib, pazopanib, and cabozantinib).^[3,4]^

TKI hepatotoxicity mechanisms involve the formation of a reactive intermediate via metabolism through the cytochrome P450 (CYP) pathway, accompanied by immune-mediated injury, impaired hepatic bile acid transport, and mitochondrial dysfunction.^[3,5]^ The most common histological finding is hepatocellular necrosis, with or without lymphocyte infiltration.^[3,5]^ Some TKIs, such as pazopanib and sorafenib, can also inhibit UDP glucuronosyltransferase isoform 1A1 (UGT1A1), causing mild and often clinically irrelevant unconjugated hyperbilirubinaemia.^[3,6]^

Immune checkpoint inhibitors (ICIs) have expanded treatment options and increased survival in specific patients with mRCC.^[7,8]^ Although they improve tumor control, they can potentially cause autoimmune pathology, as bystander non-tumor host cells are also affected. These are known as immune-related adverse events (irAEs), which when they occur in the gastrointestinal tract/liver, may result in diarrhea, colitis or hepatitis.^[9–11]^ In a meta-analysis that included 21 trials (11,454 patients), patients assigned to ICI treatment, including cytotoxic T-lymphocyte-associated protein 4 (CTLA-4), programmed cell death 1 (PD-1), and programmed cell death ligand 1 (PD-L1) inhibitors, presented with more all-grade AST elevation (Relative risk [RR] 1.80, *P* = .020) compared with non-ICI arms. Rates of high-grade AST elevation (RR 2.79; *P* = .014) were also higher in the ICI arms; the incidence of fatal irAEs was < 1%.^[12]^ The most common histological feature of ICI-induced hepatic injury is that of an acute hepatitis (autoimmune hepatitis-like) pattern of injury.^[11,13]^

In recent years, studies have been conducted on different combination therapies (different ICIs or ICIs with TKIs), some of which have already been published, showing improved efficacy outcomes but raising concerns about their safety profile.^[8,14]^

At this time in clinical practice, it is common to have to treat patients who have received different types of potentially hepatotoxic drugs. Little is known about the safety profile of sequential combination treatments with ICIs and TKIs, and whether these different types of therapies influence each other in relation to the incidence of adverse effects.

Here we report three different cases of hepatitis induced by VEGFR-TKI after receiving ICIs that present unusual characteristics in relation to what was classically observed in the pre-ICI era.

### 1.1 Case 1: Hyper-acute hepatitis with TKI after ipilimumab-nivolumab

The first case was a 77-year-old man with a previous history of high blood pressure, dyslipidemia, diabetes mellitus and chronic coronary heart disease, stable for the last 5 years.

In 2011, the patient underwent radical nephrectomy for a localized clear-cell RCC tumor. In 2016, he presented stage IV RCC with metastases to the lungs and pancreas, and was treated with sunitinib for 16 months. Due to new progressive bone disease, he received ipilimumab and nivolumab. In June 2018 (7 weeks after ipilimumab was introduced), he was admitted to hospital because of immune-related grade 3 colitis and grade 2 liver enzyme elevation, for which he was treated with high-dose steroids. Hepatic impairment improved in subsequent laboratory tests, and the steroid dose was tapered until discontinuation.

**Figure 1:**
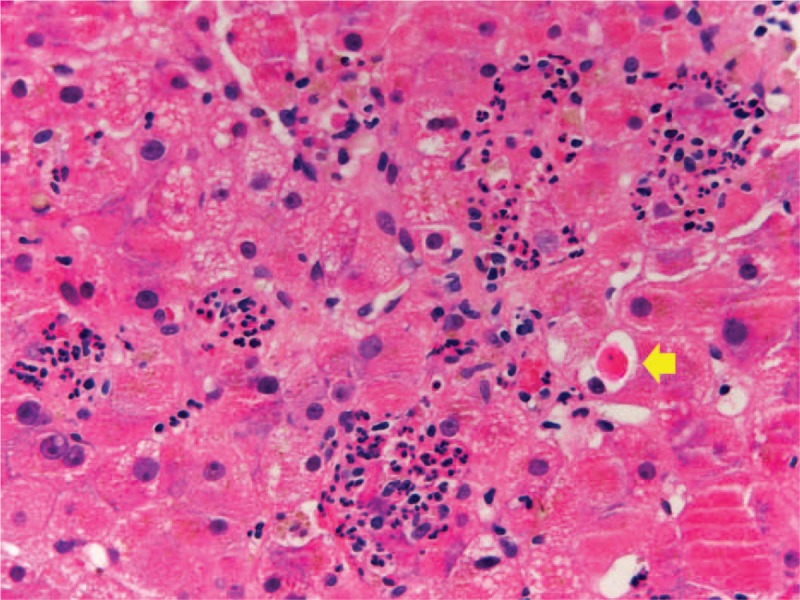
Active lobular hepatitis with infiltration of neutrophils forming microabscesses, and presence of acidophilic bodies (arrow).

**Figure 2:**
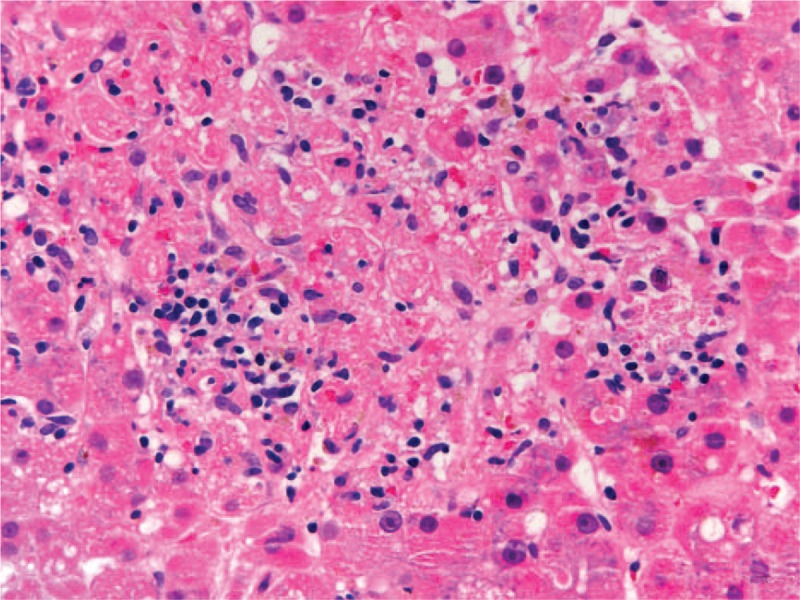
Lobular granuloma with central deposit of fibrin.

In August 2018, due to progressive disease in the lungs and bone, the patient was started on a combination of lenvatinib plus everolimus. Only 7 days after treatment was initiated, he was admitted to the emergency department with nonspecific clinical deterioration. Laboratory tests revealed grade 4 liver enzyme elevation (mainly AST and ALT elevation) with laboratory coagulopathy. Hepatic autoimmunity tests and microbiology studies were negative. Portal vein thrombosis was excluded by Doppler ultrasound. An hepatic biopsy showed lobular hepatitis with presence of granulomas (Figs. 1 and 2). These histological findings were similar to those found with the use of ICIs, so immune-related hepatitis was diagnosed in relation to the previous use of these drugs and steroid discontinuation.^[13]^ Histological lesions such as hepatocellular necrosis or granulomatous hepatitis have been also described with the use of TKIs, so the involvement of lenvatinib could not be conclusively excluded in this situation.

Lenvatinib was withdrawn and high-dose steroids were re-started. The patient improved again, enabling the steroid dose to be tapered.

Treatment with lenvatinib and everolimus was discontinued, and the patient was classified as stable disease at his last radiological evaluation.

### 1.2 Case 2: Delayed hepatitis after stopping TKI and ipilimumab-nivolumab

Case 2 involved a 60-year-old woman with a history of treated high blood pressure and right total nephrectomy due to renal lithiasis in 1980.

In 2007, left partial nephrectomy was performed for a localized clear-cell RCC. In April 2015, progressive disease was documented in the surgical site, locoregional lymph nodes and pancreas. Nivolumab combined with ipilimumab was started, but it had to be discontinued after only one cycle because of an episode of immune-related meningitis. In June 2015, the patient started second-line treatment with pazopanib (800 mg per day). After 14 days, the treatment had to be discontinued due to a potential allergic reaction (abdominal pain, exanthema and angioedema). In July 2015, two weeks after pazopanib was discontinued, the patient was admitted to hospital with elevated liver enzymes (predominantly AST and ALT elevation) with no other associated signs or symptoms. Imaging tests showed no findings of interest, and hepatic autoimmunity tests and microbiology studies were negative. Alcohol, drugs and herbal medicines were ruled out. The patient was still on a tapering course of steroids due to the previously described episode of immune-related meningitis. The abnormal liver function tests improved gradually with conservative treatment and a course of steroids; no hepatic biopsy was required.

The patient sequentially received different VEGFR-TKIs including axitinib, everolimus, sunitinib, or cabozantinib, with no other liver toxicity.

### 1.3 Case 3: Standard TKI hepatitis after nivolumab monotherapy

A 54-year-old man with no previous history of interest underwent radical right nephrectomy and adrenalectomy for a clear-cell RCC tumor in 2013.

In November 2016, after 27 months on a sunitinib/everolimus regimen, he was diagnosed with progressive disease at retroperitoneal level, for which he received nivolumab for 12 months. Due to new progression, treatment was switched to pazopanib (800 mg/day), but 7 weeks later, he presented with grade 3 liver enzyme elevation. Close monitoring showed worsening of laboratory levels with the emergence of hyperbilirubinaemia, so the patient was admitted to hospital. He had not taken alcohol or any other new drugs recently and there were no other associated signs or symptoms. Pazopanib was discontinued and a course of steroids was started; the patient responded favorably and the toxicity was resolved. No hepatic biopsy was needed. In March 2018, cabozantinib 60 mg/day was initiated, with dose reduction (40 mg/day) some weeks later due to toxicity. The patient presented with grade 1 liver enzyme elevation (AST and ALT elevation) but was able to continue on a reduced dose of cabozantinib. He is currently receiving this treatment, with radiological evidence of partial response at his last assessment.

## 2 Discussion

**Figure 3:**
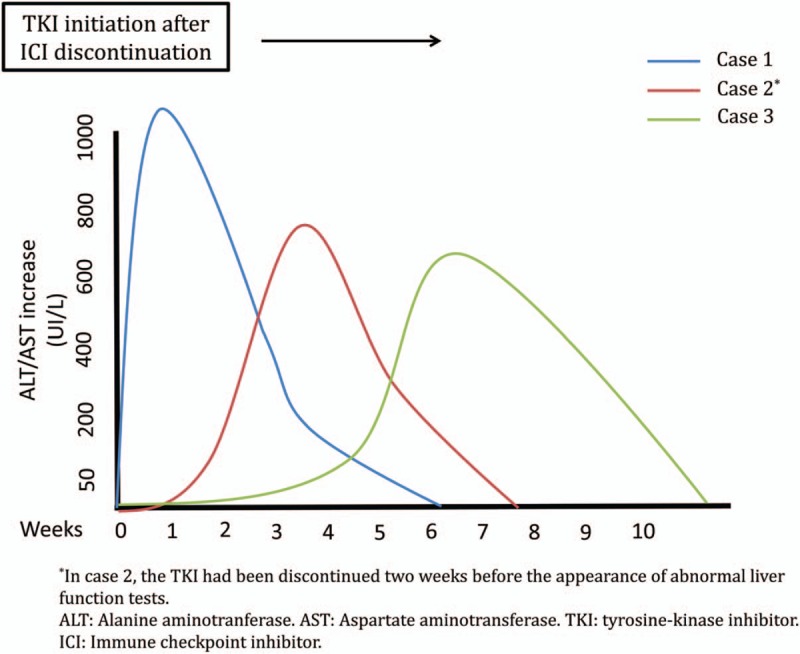
Graphic representation of the different temporal patterns.

Here we report different patterns of hepatic toxicity (mainly manifested as liver enzyme elevation) in mRCC patients treated with VEGFR-TKI after receiving nivolumab or nivolumab/ipilimumab (Fig. 3). So far, hepatic toxicity from TKIs was a well-known adverse effect usually presented as a progressive liver enzyme alteration within the first two months of therapy.^[3]^ The introduction of ICIs in the therapeutic landscape of mRCC could potentially be influencing the safety profile of TKIs given sequentially or concurrently, especially after immunotherapy combination such as nivolumab plus ipilimumab. Here we present two cases of hepatic injury after nivolumab plus ipilimumab combination, with different characteristics between them not fully understood at this moment emphasizing the possibility of toxic exacerbation with the presentation of a hyper-acute case. Those patients treated with immunotherapy in monotherapy seem to be not so compromised when given TKIs as presented in a third case.

In a meta-analysis of 18,282 patients from 52 randomized controlled trials, patients receiving VEGFR-TKIs were compared with patients not treated with these drugs.^[4]^ The RRs for high-grade liver enzyme elevations were 1.66, 1.61, 1.02, and 1.34, respectively, and the incidence of hepatic failure with VEGFR-TKI was 0.8% overall. However, there is little evidence on the potential implications of receiving TKIs after ICIs.

The pattern of hyper-acute hepatitis is particularly alarming, since liver function tests are generally checked every two weeks in patients receiving standard VEGFR-TKIs. ICIs may induce autoimmune exacerbation in different organs, including the liver, but the mechanisms by which TKI might cause hepatic injury are potentially multiple, overlapping and not fully understood. It is worth noting that an immune-mediated factor could also influence the hepatic toxicity of TKIs.

The immune system can be involved in hepatic toxicity related to both types of drugs (TKIs and ICIs), and could explain the increased risk of this adverse event in patients treated with these two therapeutic alternatives, compared to those treated with only one of these drugs.

Because of the late-onset immune-mediated events described with ICIs, patients treated with TKIs after receiving ICIs could be at higher risk of hepatic adverse events as a consequence of a synergistic effect between the drugs. This could be extremely relevant, because the immune involvement would require steroids for treatment. Thus, an additive effect must be considered by which ICIs could induce hepatic damage and later increase the risk of hepatic toxicity due to other mechanisms (not imperatively related to the immune system) associated with TKIs. The only biopsied case of our series showed findings suggestive of autoimmune hepatitis. In this regard, as mentioned above, it is also important to highlight differences between immunotherapy as monotherapy (nivolumab) or in combination (ipilimumab plus nivolumab); increased hepatic toxicity has been described with this combination and possibly with anti-CTLA-4 antibodies, compared to anti-PD-1/PD-L1 antibodies.^[15]^ In our cases, unexpected toxicities appeared after the combination treatment, while the hepatitis after nivolumab seems to be similar to the standard TKI hepatitis (Fig. 3; Table 1). This field is moving so rapidly with new combination treatments that evidence on toxicities due to cumulative effects is lacking.

**Table 1:**
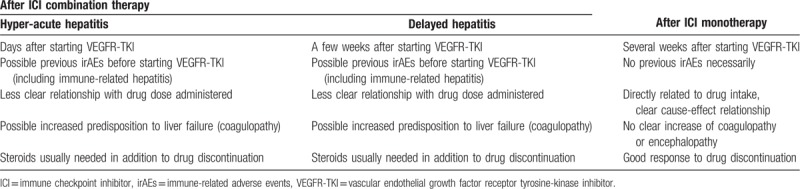
Potential patterns of short-term hepatitis in patients receiving VEGFR-TKI after ICI therapy.

In this sense, new studies assessing the efficacy and toxicity of concurrent treatment with TKIs and ICIs are also providing information about the potential increase in different adverse events when combining these drugs. Specifically, the combinations of axitinib (TKI) plus avelumab or pembrolizumab (ICI) in previously untreated mRCC do not seem to substantially increase the rate of hepatic toxicity in contrast to data described for other TKI-ICI combinations.^[16,17]^

Treatment with TKIs in monotherapy requires periodic transaminase monitoring and treatment interruption/discontinuation for specified threshold elevations of ALT. The risk of hepatic toxicity may potentially increase with the use of TKI and ICI in the same patient (concurrently or sequentially). Additional measures should be considered, specifically after the combination of CTLA-4 and PD-1 therapy. Closer adverse event monitoring, adequate patient selection (noting any history of hepatic comorbidities or hepatic metastases), and consideration of the safety profile of the different drugs (avoiding TKIs or ICIs with the highest rates of hepatic adverse events) are some factors that could help to optimize treatment in the near future. Until new prospective data is available, physicians should be aware of additional factors that may predispose to hepatitis in patients receiving ICIs.

## Author contributions

**Conceptualization:** Alberto Carretero González, Guillermo de Velasco.

**Data curation:** Alberto Carretero González, Guillermo de Velasco.

**Formal analysis:** Guillermo de Velasco.

**Investigation:** Alberto Carretero González, Guillermo de Velasco.

**Methodology:** Alberto Carretero González, Guillermo de Velasco.

**Project administration:** Alberto Carretero González.

**Resources:** Alberto Carretero González, Javier Salamanca Santamaría, Guillermo de Velasco.

**Supervision:** Alberto Carretero González, Daniel Castellano, Guillermo de Velasco.

**Validation:** Alberto Carretero González, Javier Salamanca Santamaría, Daniel Castellano, Guillermo de Velasco.

**Visualization:** Alberto Carretero González, Javier Salamanca Santamaría, Daniel Castellano, Guillermo de Velasco.

**Writing – original draft:** Alberto Carretero González.

**Writing – review & editing:** Alberto Carretero González, Daniel Castellano, Guillermo de Velasco.

Alberto Carretero González orcid: 0000-0002-2891-503X.
